# Beta-trace protein levels in epidural fluid and cerebrospinal fluid: a comparative analysis: Discriminative ability based on receiver operating characteristic analysis

**DOI:** 10.1186/s40981-026-00866-4

**Published:** 2026-06-23

**Authors:** Misuzu Hayashi, Kota Kamizato, Manabu Kakinohana

**Affiliations:** Department of Anesthesiology, University of the Ryukyus Hospital, 1076 Kiyuna, Ginowan, Okinawa 901-2725 Japan

**Keywords:** Epidural anesthesia, Cerebrospinal fluid, Beta-trace protein, Glucose test, Biomarker, Receiver operating characteristic analysis, Cerebrospinal fluid leakage

## Abstract

**Background:**

Distinguishing cerebrospinal fluid (CSF) from epidural fluid is important when the origin of aspirated fluid is uncertain during neuraxial procedures. We evaluated the discriminative ability of β-trace protein (BTP).

**Methods:**

Epidural samples were obtained via epidural catheters after local anesthetic administration, and CSF samples during spinal anesthesia. Glucose, total protein, and BTP were measured, and receiver operating characteristic (ROC) analyses were performed.

**Results:**

BTP levels were markedly higher in CSF than in epidural samples, with no overlap. Glucose overlapped between groups, whereas total protein showed limited differences. ROC analysis demonstrated excellent discrimination for BTP (AUC = 1.000) under the present study conditions, outperforming glucose (AUC = 0.904) and total protein (AUC = 0.633). For glucose, the optimal cutoff was 56.5 mg/dL (sensitivity 83.3%, specificity 90.0%). For BTP, any cutoff between 0.42–4.03 mg/L achieved 100% sensitivity and specificity within this dataset.

**Conclusions:**

BTP shows strong discrimination between CSF and epidural fluid.

**Supplementary Information:**

The online version contains supplementary material available at https://doi.org/10.1186/s40981-026-00866-4.

## Background

Epidural anesthesia and spinal anesthesia are both widely used techniques for perioperative analgesia and anesthesia. In clinical practice, it may be difficult to determine whether fluid obtained through a needle represents cerebrospinal fluid (CSF), particularly when performing spinal anesthesia after epidural analgesia, where clear fluid may be encountered and misidentified as CSF [1–3]. In such situations, residual epidural injectate or other fluids may mimic CSF, leading to diagnostic uncertainty and potential failure of spinal anesthesia. Furthermore, in case of unintended migration of an epidural catheter into the subarachnoid space, it is necessary to determine whether the aspirated fluid represents CSF or residual administered fluid. Accurate identification is clinically important, as misinterpretation may result in inappropriate catheter removal, unsafe continuation of epidural administration [4, 5], or ineffective spinal anesthesia [1, 3].

The glucose test has traditionally been used to differentiate CSF from epidural aspirates, which may contain saline or local anesthetics administered into the epidural space [6–9]. However, previous studies have shown that glucose concentrations in epidural aspirates may vary over time after local anesthetic administration and may gradually approach those of CSF, leading to potential false-positive results and limiting its reliability [10, 11]. Therefore, a clinically useful biomarker should be robust to such temporal variation, and a more specific marker is needed to improve diagnostic accuracy. 

Beta -trace protein (BTP), also known as lipocalin-type prostaglandin D synthase, is a protein that is highly concentrated in CSF compared with other body fluids [12–14]. It has been investigated as a marker for detecting CSF leakage in otolaryngological and neurological settings [15–19]. However, its characteristics in epidural samples remain poorly defined.

Therefore, as an initial step, we aimed to characterize the behavior of BTP in epidural aspirates under controlled conditions. The present study aimed to compare BTP concentrations between epidural aspirates and CSF samples and to evaluate their discriminative ability using receiver operating characteristic analysis, and to assess the temporal stability of these biomarkers in relation to the time elapsed after epidural administration.

## Methods

### Study design and ethical approval

This prospective observational study was approved by the Ethics Committee of the University of the Ryukyus for Medical and Health Research Involving Human Subjects (approval nos. 1516 [November 2019] and 1545 [January 2020]). Written informed consent was obtained from all participants.

### Patients and sample collection

#### Study 1: Sample collection from epidural space

Patients with American Society of Anesthesiologists physical status (ASA-PS) I or II, aged 20–75 years, undergoing elective surgery under general anesthesia combined with epidural analgesia between November 2019 and April 2020 were included. Patients with cerebrospinal disease or dural puncture were excluded.

The epidural catheterization procedures were performed using our standard methods. In summary, an epidural catheter (19-gauge Perifix®; B.Braun, Germany) was placed using an 18-gauge Tuohy needle using a loss-of-resistance technique with saline in the left lateral decubitus position. After confirming no aspiration of fluid, a test dose of 2 mL of 1% lidocaine was administered to exclude subarachnoid placement.

**Fig. 1:**
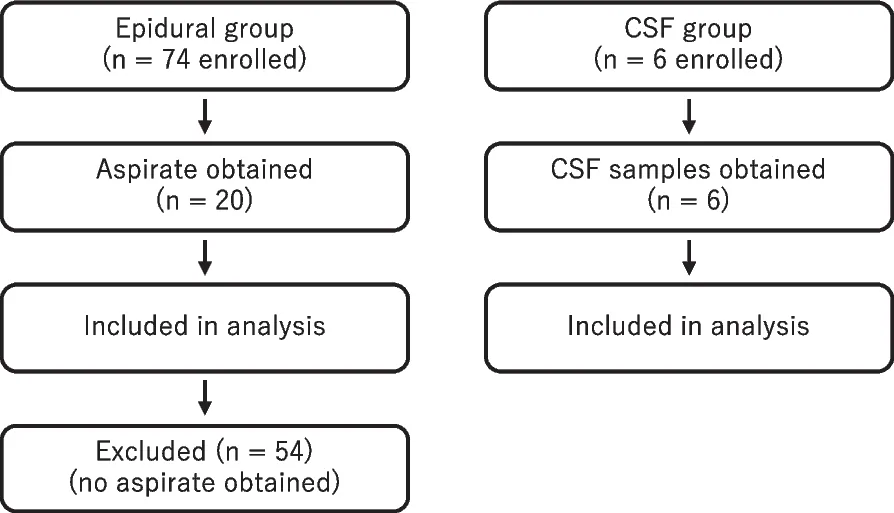
Flow diagram of patient enrollment. A total of 74 patients were enrolled for epidural sampling, and aspirates were obtained from 20 patients. Fifty-four patients were excluded due to inability to obtain aspirate samples. In the CSF group, all 6 enrolled patients provided CSF samples and were included in the analysis

General anesthesia was induced and maintained, and local anesthetics were administered via the epidural catheter for intraoperative analgesia. The choice of local anesthetic was at the discretion of the attending anesthesiologist (details shown in Supplementary Table 1). More than 15 min after the last epidural administration, aspiration was attempted. When fluid could be aspirated, the initial 0.2 mL was discarded as dead space within the catheter, and an additional 0.3 mL sample was subsequently collected for analysis. Patients from whom no aspirate could be obtained were excluded. Enrollment was continued until 20 epidural samples were obtained. The patient flow is shown in Fig. 1.

#### Study 2: Sample collection from subarachnoid space

Six patients with ASA-PS I or II, aged 20–75 years, undergoing elective surgery under spinal anesthesia were enrolled. After confirmation of cerebrospinal fluid reflux through a 25-gauge spinal needle (Spinocan® Quincke; B. Braun), 0.3 mL of CSF was collected before anesthetic injection.

### Sample processing and biological analysis

From each sample, 0.3 mL was obtained. Of this, 0.2 mL was aliquoted into microtubes and stored at −80℃ for later analysis, while the remaining 0.1 mL was immediately used for glucose measurement using a blood gas analyzer (ABL825 FLEX; Radiometer, Denmark).

Total protein concentrations were measured as a comparative marker to BTP using a mDrop plate (Thermo Fisher Scientific, USA). Absorbance was measured with a plate reader (Multiskan Sky®, Thermo Fisher Scientific, USA), and concentrations were calculated according to the manufacturer’s instructions.

BTP concentrations were measured using a commercially available enzyme-linked immunosorbent assay (ELISA) kit (Human Prostaglandin D Synthase [lipocalin-type]; BioVendor, Czech Republic). Epidural samples were diluted 1:100 based on preliminary testing, and CSF samples were diluted 1:500 according to the manufacturer’s instructions.

Clinical data including patient characteristics (Table 1), puncture level, type of surgery, local anesthetic administered (Supplementary Table 1), and time from epidural drug administration to sample collection were recorded.

**Table 1:** Patient characteristics

	Epidural aspirate (*n* = 20)	CSF (*n* = 6)
Sex (M/F)	3/17	1/5
Age (years)	52.4 ± 13.9	42.8 ± 12.6
Height (cm)	155.9 ± 5.6	156.3 ± 5.5
Weight (kg)	54.5 ± 7.5	65.3 ± 14.9
ASA-PS (I/II)	6/14	3/3
Puncture Level (n)		
Lower Thoracic (Th7-Th12)	20	0
Lumbar (L3-L5)	0	6
Type of surgery (n)		
Gynecology	13	5
Urology	5	0
Hepatosurgery	2	0
Dermatology	0	1

Data are expressed as mean ± standard deviation or number. Thoracic levels indicate Th7-Th12, and lumbar levels indicate L3-L5

*Abbreviations CSF* Cerebrospinal fluid, *ASA-PS* American Society of Anesthesiologists Physical Status, *Th* Thoracic vertebra, *L* Lumbar vertebra

### Statistical analyses

**Fig. 2:**
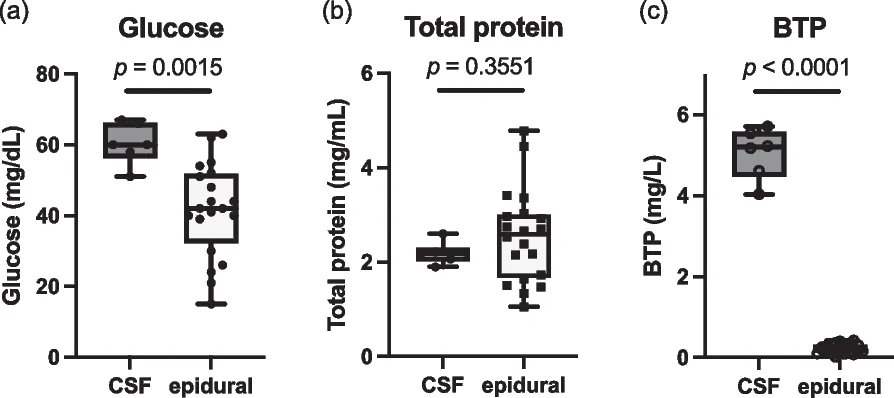
Comparison of glucose, total protein, and β-trace protein levels between cerebrospinal fluid and epidural samples. Individual data points are overlaid on box-and-whisker plots (median, interquartile range, and range). **a** Glucose concentrations were significantly higher in cerebrospinal fluid than in epidural samples. **b** Total protein concentrations did not differ significantly between the groups. **c** β-trace protein concentrations were markedly higher in cerebrospinal fluid than in epidural samples, with no overlap between groups. Statistical comparisons were performed using the Mann–Whitney test. *p* values are indicated in the figure

**Fig. 3:**
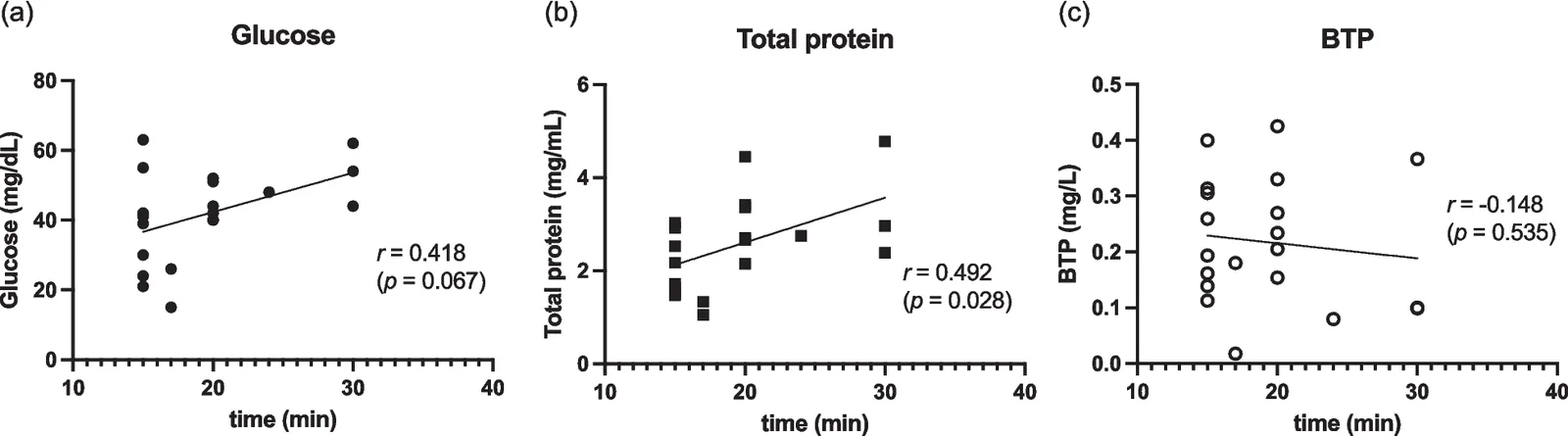
Relationship between sampling time and biomarker concentrations in epidural samples after epidural drug administration. Scatter plots show the associations between the time from epidural drug administration to sample collection and the concentrations of (**a**) glucose, (**b**) total protein, and (**c**) β-trace protein in epidural samples. Glucose concentrations tended to increase over time, whereas total protein concentrations showed a significant positive correlation with sampling time. In contrast, β-trace protein concentrations showed no significant temporal correlation. These findings suggest that, under the present study conditions, β-trace protein concentration in epidural samples is less affected by sampling time than glucose and total protein. Correlation coefficients (*r*) and *p* values are shown in each panel

**Fig. 4:**
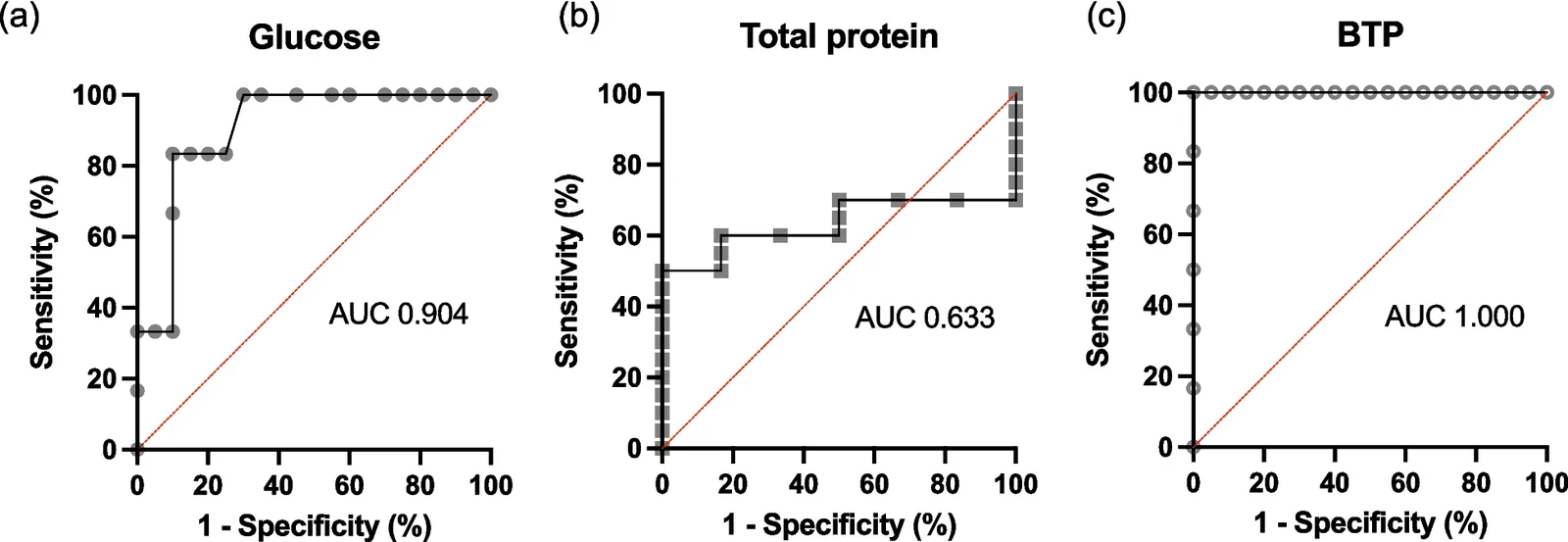
ROC curves for glucose, total protein, and β-trace protein distinguishing cerebrospinal fluid and epidural samples. ROC curves are shown for (**a**) glucose, (**b**) total protein, and (**c**) β-trace protein. The area under the curve (AUC) was 0.904 for glucose, 0.633 for total protein, and 1.000 for β-trace protein. β-trace protein demonstrated excellent discrimination under the present study conditions between cerebrospinal fluid and epidural samples

Statistical analyses were performed using GraphPad Prism ver. 10.4.1 (GraphPad Software, USA). Data are presented as mean ± standard deviation or median (interquartile range) as appropriate. Comparisons between epidural and CSF samples were performed using the Mann–Whitney test (Fig. 2). Correlations between sampling time and biomarker concentrations were assessed using Spearman’s correlation coefficient (Fig. 3). Receiver Operating Characteristic (ROC) analysis was conducted to evaluate the discriminative ability of each biomarker, and the area under the curve (AUC) was calculated (Fig. 4). The optimal cutoff value was determined using the Youden index. A *p* value < 0.05 was considered statistically significant.

## Results

### Patient characteristics

A total of 74 patients were enrolled for sampling from epidural space, of whom aspirates were successfully obtained from 20 patients (Study 1). Fifty-four patients were excluded due to inability to obtain aspirate samples. In the CSF group, all 6 enrolled patients provided CSF samples (Study 2). The patient flow is shown in Fig. 1.

The characteristics of the patients included in the analysis are summarized in Table 1. Epidural catheter placement was performed at the thoracic level in all patients in the epidural group, whereas CSF samples were obtained from lumbar puncture.

### Comparison of biomarker concentrations

BTP concentrations were markedly higher in CSF than in the epidural samples, with no overlap between the groups (Fig. 2c). In contrast, glucose concentrations overlapped between the groups (Fig. 2a), and total protein did not differ significantly between the groups (Fig. 2b).

### Temporal changes in biomarker concentrations

The relationships between sampling time and biomarker concentrations are shown in Fig. 3. Glucose and total protein concentrations showed positive correlations with time after epidural drug administration, whereas BTP concentrations showed no significant correlation.

### Discriminative ability of biomarkers

Receiver operating characteristic curves are shown in Fig. 4. The AUC was 1.000 for BTP, indicating excellent discrimination between CSF and epidural samples. In comparison, glucose showed good discriminative ability (AUC = 0.904), whereas total protein showed limited discriminative ability (AUC = 0.633). The optimal cutoff value for glucose was 56.5 mg/dL, yielding a sensitivity of 83.3% and a specificity of 90.0%. For BTP, no overlap was observed between groups. Therefore, any cutoff value between the highest epidural value and the lowest CSF value (0.42–4.03 mg/L) would achieve 100% sensitivity and specificity under the present study conditions.

### Epidural drug administration

The types and volumes of local anesthetics administered via the epidural catheter are summarized in Supplementary Table 1.

### Adverse events

No patients in the epidural group showed signs of unintended spinal block. One patient reported headache on postoperative day 1, which resolved spontaneously.

## Discussion

The present study demonstrated that β-trace protein (BTP) levels were markedly higher in cerebrospinal fluid (CSF) than in epidural samples, with no overlap between the groups. In contrast, glucose concentrations showed overlapping distributions, and total protein demonstrated limited discriminative ability. Receiver operating characteristic analysis confirmed that BTP achieved excellent discrimination (AUC = 1.000) under the present controlled study conditions, highlighting its potential clinical utility and outperforming conventional markers.

These findings may be explained by the biological characteristics of BTP. BTP is highly concentrated in CSF and present at markedly lower levels in peripheral compartments [12]. In contrast to small molecules such as glucose, which readily diffuse across biological membranes and may be influenced by local conditions, BTP appears to remain relatively compartment-specific. This characteristic likely contributes to the clear discrimination observed between CSF and epidural samples.

Temporal changes in biomarker concentrations also provide important insights into their clinical utility. A previous study reported that glucose levels in epidural aspirates increase over time when repeatedly sampled from the same patient [11]. Although only a single sample was obtained per patient in the present study, the observed time-dependent increase in glucose is consistent with this finding. Total protein also showed a positive correlation with time, suggesting similar time-dependent processes within the epidural space, such as diffusion or local physiological changes. In contrast, BTP concentrations showed no significant correlation with sampling time, indicating relative stability. This time-independent characteristic further supports the robustness of BTP as a biomarker for distinguishing CSF from epidural fluid, particularly in clinical situations where the timing of sampling is variable or uncertain.

From a clinical perspective, conventional methods, such as testing with fluid glucose levels, may be unreliable. Although glucose concentrations differ significantly between groups, there is considerable overlap between epidural aspirates and CSF, limiting its clinical utility for differentiation. The present findings suggest that BTP may provide a more reliable indicator for differentiating these fluids. However, at present, BTP measurement is primarily performed using laboratory-based methods, which are not suitable for rapid decision-making in clinical settings. ELISA-based measurement of BTP typically requires several hours and is therefore not suitable for immediate intraoperative decision-making. Recent advances in lateral flow immunoassay technologies may enable rapid point-of-care detection of BTP in the future, although such devices are not yet widely available for routine clinical use [17, 18].

Previous studies have demonstrated that BTP is a useful marker for identifying CSF leakage in various clinical settings, such as suspected CSF rhinorrhea [15, 17, 19] or post-spinal surgery CSF leakage [18]. However, its characteristics in epidural samples have not been fully elucidated. The present study extends these findings by demonstrating a clear distinction between epidural and CSF samples, supporting its potential utility in this context.

A strength of this study is the direct comparison of epidural and CSF samples under controlled clinical conditions, along with the use of ROC analysis to quantitatively evaluate discriminative ability. The present study should be considered as a preliminary step to establish the baseline behavior of BTP in epidural aspirates, which is necessary before evaluating its clinical utility across specific clinical scenarios.

This study has several limitations. First, the sample size was relatively small, which may limit the generalizability of the findings. Second, epidural samples were obtained only from cases in which aspirates were available, which may introduce selection bias.

Third, the experimental conditions do not fully reflect the most clinically challenging scenarios. In the present study, epidural samples were obtained under controlled conditions, and CSF samples consisted of CSF collected during spinal anesthesia. Therefore, the study does not directly evaluate equivocal or mixed samples, such as those encountered in cases of accidental dural puncture, CSF leakage into the epidural space, or intrathecal catheter migration. In such situations, fluid composition may be more complex, and the diagnostic performance of BTP remains to be established. Accordingly, the present findings should be interpreted as demonstrating the baseline discriminative potential of BTP rather than its definitive clinical performance in ambiguous samples.

Fourth, epidural samples were obtained at the thoracic level, whereas CSF samples were collected from the lumbar region. Previous studies have demonstrated that BTP exhibits a rostrocaudal concentration gradient within the CSF, with higher concentrations observed in lumbar CSF compared with ventricular CSF [12]. However, BTP concentrations at intermediate levels, such as the thoracic region, remain unclear. Therefore, a potential regional difference cannot be excluded, although its impact on the present findings is likely limited given the clear separation observed between groups.

Finally, although BTP showed excellent discriminative performance, its current clinical applicability is limited by the lack of rapid point-of-care testing methods. While recent advances in lateral flow immunoassay technologies may enable rapid bedside detection in the future, such devices are not yet widely available for routine clinical use.

Therefore, further studies are warranted to validate the clinical applicability of BTP in more complex and clinically relevant settings.

## Conclusions

In conclusion, BTP shows strong discriminative ability between CSF and epidural fluid and may serve as a robust and clinically useful marker when the origin of the fluid is uncertain.

## Supplementary Information

Supplementary Material 1: Supplementary Table 1. Content and volume of local anesthetics administered via the epidural catheter before sample collection.

## Data Availability

The datasets generated and/or analyzed during the current study are not publicly available due to privacy considerations but are available from the corresponding author on reasonable request.

## Abbreviations

BTP: β-trace protein
CSF: Cerebrospinal fluid
ROC: Receiver operating characteristic
AUC: Area under the curve
ASA-PS: American Society of Anesthesiologists Physical Status
ELISA: Enzyme-linked immunosorbent assay
