# Supplementary material for: Beta-trace protein levels in epidural fluid and cerebrospinal fluid: a comparative analysis: Discriminative ability based on receiver operating characteristic analysis
Source: JA Clin Rep. 2026 Jun 23;12:30. doi: 10.1186/s40981-026-00866-4 (PMC13542016; doi:10.1186/s40981-026-00866-4)
Supplement: Supplementary file 1 [file 40981_2026_866_MOESM1_ESM.docx]

**Supplementary Table 1** Content and volume of local anesthetics administered via the epidural catheter before sample collection

| Local anesthetics | Concentration | n |
| --- | --- | --- |
| Lidocaine | 0.2% | 2 |
|  | 1% | 1 |
| Ropivacaine | 0.125% | 1 |
|  | 0.15% | 8 |
|  | 0.2% | 1 |
|  | 0.25% | 3 |
|  | 0.75% | 1 |
| Levobupivacaine | 0.17% | 1 |
| Saline | - | 2 |
| Volume (mL): 10 (6.7-10.7) | | |

Data are expressed as number or median (interquartile range).
